# Drug‐Induced Cauda Equina Syndrome in an 8‐Year‐Old Boy With Acute Lymphoblastic Leukemia: An Uncommon Case Report

**DOI:** 10.1002/ccr3.70122

**Published:** 2025-01-16

**Authors:** Marzieh Babaee, Mohsen Javadzadeh, Ali Hazeghi

**Affiliations:** ^1^ Physical Medicine and Rehabilitation Research Center Shahid Beheshti University of Medical Sciences Tehran Iran; ^2^ Pediatric Neurology Department, Pediatric Neurology Research Center Shahid Beheshti University of Medical Sciences Tehran Iran

**Keywords:** cauda equina syndrome, methotrexate, pediatrics, precursor cell lymphoblastic leukemia‐lymphoma

## Abstract

Intrathecal methotrexate can cause cauda equina syndrome in pediatric ALL patients, as demonstrated in this rare case of an 8‐year‐old boy. Symptoms included progressive limb weakness and urinary retention. Early recognition, prompt discontinuation of the offending agent, and multidisciplinary management are crucial. Vigilant neurological monitoring is essential for pediatric acute lymphoblastic leukemia patients receiving intrathecal chemotherapy.

**Abbreviations**
ALL: acute lymphoblastic leukemia
CMAPs: compound muscle action potentials
CNS: central nervous system
CSF: cerebrospinal fluid
IT: intrathecal
MMT: manual muscle testing
MRI: magnetic resonance imaging
MTX: methotrexate

## 1 Introduction

Acute lymphoblastic leukemia (ALL) is the most common childhood malignancy. Its treatment often involves the use of intrathecal chemotherapeutic agents such as methotrexate (MTX), cytarabine, and hydrocortisone [1]. While intrathecal chemotherapy is effective, it can occasionally lead to various central nervous system (CNS) complications, including arachnoiditis, seizures, encephalopathy, and spinal cord lesions [2]. In pediatric patients, it is possible that the incidence of neurological complications in this setting is underestimated because cases may go unrecognized or unreported [3]. A review of neurological manifestations of ALL in children found symptoms in 9.5% (90/923) patients aged 1–11 years, with more prominent seizure and encephalopathy [4].

Although the cauda equina syndrome was reported more in the adult group, it is a less reported side effect after intrathecal (IT) chemotherapy [2, 5]. Cord lesions primarily occur with intrathecal MTX and Ara‐C combinations or high‐dose/slow‐release Ara‐C alone. These lesions often cause permanent motor deficits, bowel, and bladder dysfunction. The onset of neurotoxicity following intrathecal chemotherapy administration typically occurred within a mean of 10 days, with a latency period ranging from 1 to 91 days [6]. Common symptoms include severe bowel and bladder dysfunction, accompanied by varying degrees of muscle weakness. Sensory impairments are uncommon. Magnetic resonance imaging (MRI) is a valuable tool for differentiating neurotoxicity caused by intrathecal chemotherapy from other spinal cord conditions, such as tumor‐related compression [6].

Of the 29 reported cases with spinal cord lesions after IT chemotherapy, cauda equina syndrome occurred in three cases, which showed poor recovery and are left with residual neurological deficits [6]. Meanwhile, this side effect has not been reported in children after IT chemotherapy.

We present a rare case of drug‐induced cauda equina syndrome that occurred following intrathecal chemotherapy in an 8‐year‐old boy with ALL. This highlights differences in patient age and symptoms compared to prior reports. It underscores the need for vigilant neurological monitoring in pediatric ALL patients receiving intrathecal chemotherapy, particularly methotrexate and cytarabine.

## 2 Case Presentation/Examination

An 8‐year‐old boy diagnosed with pre‐B cell ALL had been undergoing chemotherapy for 1 year, comprising intravenous vincristine at weekly intervals and intrathecal MTX (12.5 mg) at monthly intervals. He exhibited neurological symptoms 2 weeks after completing his twelfth session of intrathecal chemotherapy. The initial symptom observed was paresthesia of the feet, which was soon followed by limb pain, limb weakness, persistent urinary retention, and fecal incontinence. The patient showed no improvement after receiving IVIG (2 g/kg) treatment at another center. Consequently, the patient was admitted to our hospital for further evaluation and management.

### 2.1 Clinical Examination

Clinical examination at the time of admission revealed bilateral, symmetrical lower limb weakness. Manual muscle testing (MMT) revealed a score of 1/5 in both proximal and distal regions of the lower limbs and 4/5 in the upper limbs. Deep tendon reflexes (DTR) testing showed no response in bilateral knees and ankles. No pathological reflexes, like the Babinski sign or ankle clonus, were elicited. The patient had urinary retention. No sensory level was identified. No signs of cerebellar ataxia, extrapyramidal movements, or local spine tenderness were observed. Detailed neuromuscular examination revealed a neurogenic lesion significantly affecting the bilateral L5 myotomes. Based on these clinical features, a diagnosis of hematoma or thrombosis was considered.

## 3 Methods

### 3.1 Diagnostic Investigations

**FIGURE 1:**
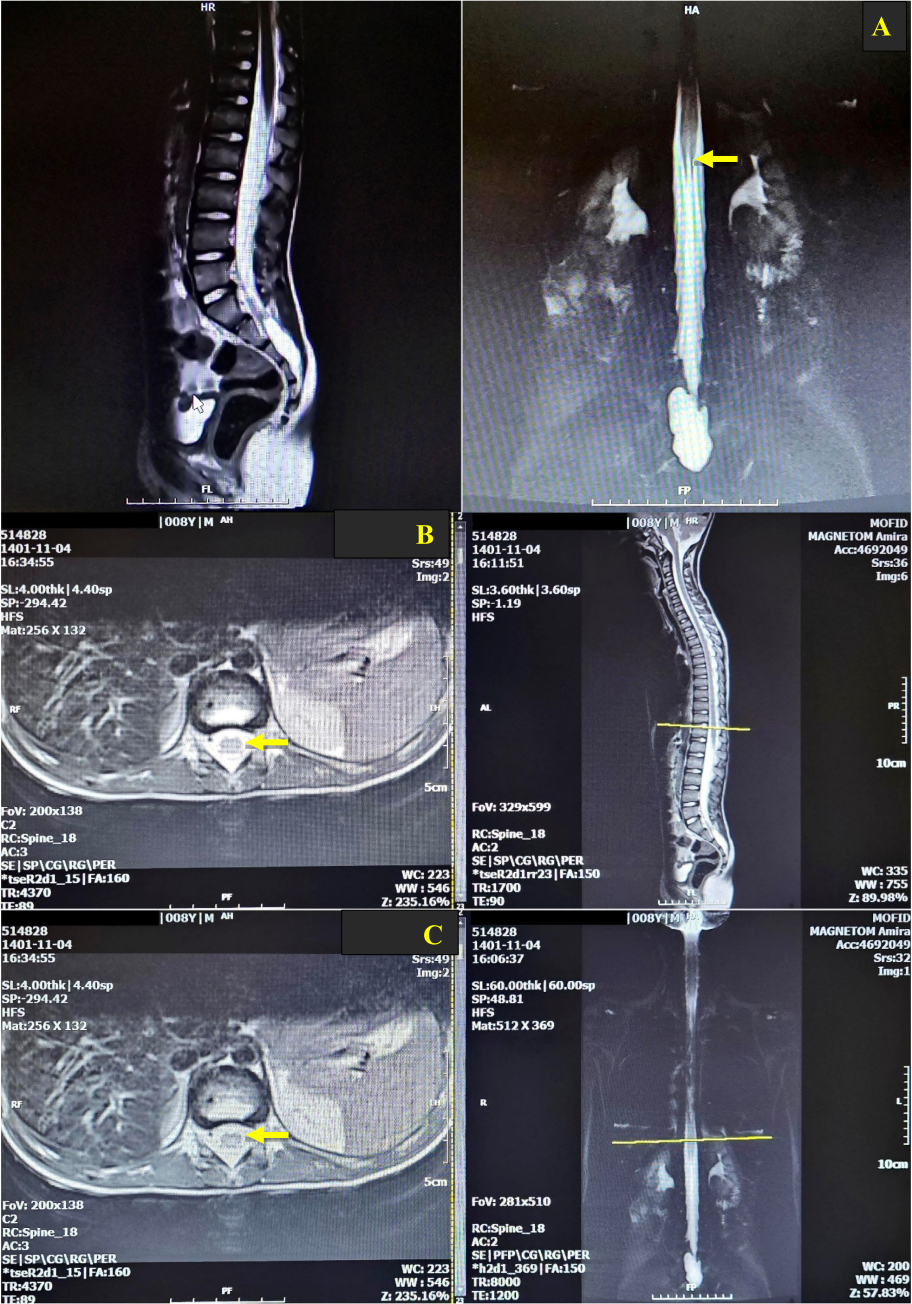
MRI of spine with increased enhancement in the cauda equina region, particularly in the ventral roots (A–C).

Pre‐chemotherapy lumbar puncture showed normal cerebrospinal fluid (CSF) glucose (56 mg/dL; normal range: 50–80) and protein (20 mg/dL; normal range: 12–45) levels, and the gram stain and culture of the CSF yielded negative results for microbial agents. Besides, there were no findings of white blood cells or malignant cells in the CSF. Additionally, the patient's laboratory test results indicated a hemoglobin level of 14 g/L (normal range for this age: 11.5–15.5), erythrocyte sedimentation rate (ESR) of 26 mm/h (normal for age < 50 years: 20 mm/h): aspartate aminotransferase (AST) of 40 IU/L (normal range: female: up to 31, male: up to 37), alanine aminotransferase (ALT) of 71 IU/L (normal range: female: up to 31, male: up to 41), alkaline phosphatase (ALKP) of 46.3 IU/L (normal range: 180–1200), and C‐reactive protein within the normal range (5 g/L) (normal range: up to 10). Lumbosacral MRI revealed a 42 × 10 mm Tarlov cyst within the sacral canal with increased enhancement in the cauda equina region, particularly in the ventral roots. Brain, neck, and thoracic MRI revealed no evidence of abnormality (Figure 1).

Nerve conduction study and electromyography (EMG/NCS) findings indicated low‐amplitude or absent compound muscle action potentials (CMAPs) in the lower limbs, while other sensory nerve action potentials (SNAPs) and CMAPs remained within the lower limit of normal. The electrodiagnostic study findings were consistent with bilateral lumbosacral polyradiculopathy, from L2 to S1, with more severity in L5.

### 3.2 Differential Diagnosis

The clinical and radiological findings raised several differential diagnoses, including Guillain–Barré syndrome, MTX‐induced neuropathy, and CNS involvement from ALL. Guillain–Barré syndrome was subsequently excluded by electrodiagnostic study. The patient was diagnosed with cauda equina syndrome based on his clinical presentation, the results of the electrodiagnostic study, and imaging findings.

### 3.3 Treatment and Management

Intrathecal MTX was promptly discontinued. Supportive treatment was provided, and intravenous pulse methylprednisolone (500 mg daily for 5 days) was added. However, the patient showed no clinical improvement. He received a Foley catheter insertion, as he did not have any urination sense and was unable to void at first. However, a significant improvement in urinary function was observed during follow‐up after intravenous pulse methylprednisolone therapy. He received a rehabilitation program focused on wheelchair mobility, upper limb strengthening, activities of daily living, and management of neurogenic bladder and bowel dysfunction.

After 6 months of rehabilitation, the patient achieved walking with some difficulty. However, at 1‐year follow‐up, he exhibited impaired balance during walking (clumsy gait) and bilateral foot drop. Mental status and upper limb function were normal.

## 4 Conclusions and Results

MTX neurotoxicity can affect both the central and peripheral nervous systems, with an incidence of 3%–11% in children [2]. However, cauda equina syndrome due to intrathecal MTX injection was less reported in children. Several mechanisms for the adverse effects of intrathecal MTX have been proposed. MTX can selectively damage motor nerves, differing from immune‐mediated mechanisms seen with monoclonal antibodies [1, 7]. The neurotoxic effects of intrathecal MTX involve folate metabolism in the spinal cord, particularly in the dorsal columns and motor neurons. Further, exposure to a high dose of intrathecal MTX in the long term can alter neural tissues. Other theories suggest that methyl hydroxybenzoate and propyl hydroxybenzoate, which are used as preservatives, can induce conduction block of spinal nerve roots and propagate degenerative changes of nerves, respectively. The concurrent use of intrathecal MTX and intrathecal cytarabine may increase the neurotoxicity of MTX, due to the longer half‐life of cytarabine in the CSF, which interferes with DNA synthesis and cell replication in CNS cells [2, 5]. MTX neurotoxicity mechanism was investigated before the concentration of myelin basic protein in the media increased significantly after 3 weeks of MTX exposure and was higher than the control after 5 weeks. Large amounts of MTX in the CSF are concomitant with more severe neurotoxicity effect. These findings suggest that MTX primarily acts as a neuronal toxin in cerebellar explant cultures, and the demyelination observed is a consequence of axonal loss rather than a change in oligodendroglial cell function. So, disease‐modifying therapies or immunomodulatory medications may be useful in this situation [8].

Kwong et al. reviewed 28 cases with detailed neurologic information. Paralysis was the most common complication, affecting 82% of patients (paraplegia) and 11% (tetraplegia). Additionally, 7% experienced cauda equina syndrome, which occurred in patients aged 42 and 65. These cases strongly suggest that combined intrathecal MTX and Ara‐C is the primary risk factor for spinal cord lesions. When Ara‐C is used alone, high doses or slow‐release preparations, often given with systemic chemotherapy, are associated with this severe complication. Unfortunately, spinal cord damage from these treatments is often permanent, resulting in long‐term motor, bowel, and bladder problems [6].

Our patient's case differs significantly from the typical presentation of MTX‐induced neurotoxicity with cauda equina syndrome described in the literature. Firstly, the patient's young age is atypical, as most reported cases involve adults [7, 8]. The clinical presentation was also unusual, characterized by foot numbness, worsening limb weakness, and severe urinary retention, which persisted despite discontinuation of MTX and steroid treatment [9, 10]. Secondly, the onset of symptoms after the 12th chemotherapy session suggests a cumulative MTX toxicity rather than an acute reaction [11].

Evaluating such conditions requires comprehensive history and examination, along with electrodiagnostic, laboratory, and imaging studies. In previous cases, the absence of motor F‐wave response was observed as an early sign of neurotoxic damage, even before a reduction in CMAP amplitude in distal motor nerves. Similar changes in F‐waves are seen in Guillain–Barré syndrome after 4–10 days of clinical onset. Neurophysiological studies have shown specific nerve damage patterns in Guillain–Barré syndrome and lymphoproliferative disorders [3]. In this study, NCS wave abnormalities were observed. Rostral spinal cord injury led to postsynaptic changes in caudal spinal motor neurons. Progressive decrease in CMAP amplitude indicated severe axonal degeneration. Protein concentration in CSF may be present in MTX‐treated cases, but no change was found in CSF protein in this case. Gadolinium‐enhanced MRI revealed T2 hyperintensities in lumbar nerve roots and spinal cord, while thoracic vertebrae in this case appeared normal.

## 5 Discussion

Generally, neurotoxicity of intrathecal MTX may develop within days to weeks, and often has a poor prognosis. Some studies suggest that the use of normal saline or distilled water as diluents may exacerbate chemical irritation to the nerves by altering acidity levels or changing ionic contents [5]. Unfortunately, treatment options for chemotherapy‐related neurotoxicity are limited. Clinical improvement has been observed in a few cases, with the administration of methylprednisolone with folic acid and vitamin B12 supplementation. Methylprednisolone, a corticosteroid with potent anti‐inflammatory properties, may offer a therapeutic avenue for managing chemotherapy‐induced cauda equina syndrome (CES) [12, 13]. By suppressing inflammation around the compressed cauda equina nerve roots, methylprednisolone could potentially alleviate pain and improve nerve function. However, the efficacy of methylprednisolone in this context remains uncertain, with limited evidence supporting its use [3, 6, 12, 13]. Further research is necessary to definitively establish its clinical benefit and optimize treatment strategies.

Additionally, a study by Drachtman and colleagues found that dextromethorphan was effective in managing methotrexate‐related side effects in children [14]. Therefore, there is no standard recommendation due to unknown mechanisms and limited evidence from clinical trials. In summary, while MTX neurotoxicity is recognized in leukemia treatment [11, 15], this case provides unique perspectives, underscoring vigilant monitoring and diagnostic measures.

Children with ALL undergoing intrathecal chemotherapy, especially MTX, are at risk of progressive neurotoxicity. These neurological manifestations warrant vigilant monitoring for early detection and intervention. It is critical for pediatric neurologists and oncologists to promptly recognize symptoms. A collaborative approach between pediatric neurology, oncology, and physiatry is essential for optimal care. Developing guidelines or protocols at cancer centers can promote early identification and management. Routine post‐chemotherapy neurological assessments may significantly impact outcomes. Further research is needed on neuroprotective agents and chemotherapy protocol modifications to reduce neurotoxicity. Larger prospective studies will provide insights into the true incidence, risk factors, and outcomes of such complications. Additionally, the implementation of multicenter drug vigilance programs is necessary to better understand the risk factors associated with neurotoxicity and improve patient outcomes.

On the whole, vigilant neurological monitoring and a multidisciplinary approach are paramount in caring for pediatric ALL patients receiving intrathecal chemotherapy, to enable early intervention for neurotoxicity.

## Author Contributions

**Marzieh Babaee:** conceptualization, writing – review and editing. **Mohsen Javadzadeh:** conceptualization, supervision, writing – review and editing. **Ali Hazeghi:** conceptualization, writing – original draft.

## Ethics Statement

The authors have nothing to report.

## Consent

Written informed consent was obtained from the parents of the patient for the publication of any potentially identifiable images or data. The parents of the patient have given parental consent for this study.

## Conflicts of Interest

The authors declare no conflicts of interest.

## Data Availability

The main medical data are reported in the study. The original version of the medical experiments is available on request from the corresponding author.
